# Influence of Puncture Devices on the Accuracy of Cyclophosphamide Dosing for Chemotherapy Administration

**DOI:** 10.3390/ph18060879

**Published:** 2025-06-12

**Authors:** Susana Carvalho, Andreia Cardoso, Débora Ferreira, Diana Dias da Silva, Fernando Moreira

**Affiliations:** 1 REQUIMTE/LAQV, Escola Superior de Saúde, Instituto Politécnico do Porto, Rua Dr. António Bernardino de Almeida, 4200-072 Porto, Portugal; mar_ga_ri_da20@hotmail.com; 2 Centro Hospitalar Universitário São João, Alameda Prof. Hernâni Monteiro, 4200-319 Porto, Portugal; andreiasocardoso1996@gmail.com (A.C.);

**Keywords:** high-performance liquid chromatography with diode-array detection (HPLC-DAD), cyclophosphamide, anticancer drugs, chemotherapy preparation, aspiration devices, quality control, dose accuracy

## Abstract

**Background/Objectives:** Cyclophosphamide is one of the most commonly used cytotoxic drugs in chemotherapy protocols. Its preparation in the hospital setting involves handling concentrated solutions, which pose occupational exposure risks and potential variations in the final dose administered. The aim of this study was to evaluate the effect of aspiration devices on the concentration of cyclophosphamide in reconstituted solutions. **Methods**: An analytical method was validated using high-performance liquid chromatography coupled to a diode-array detector (HPLC-DAD) for quality control. Cyclophosphamide solutions were prepared and aspirated using either a conventional needle or spike device with or without a filtration system. **Results**: The validated method demonstrated linearity (R^2^ = 0.9999), high precision (0.22–4.59%) and accuracy (88.9–99.4%), with a limit of quantification of 4.03 µg/mL. Significant differences (*p* < 0.001) were observed between samples aspirated with a needle and those aspirated with a spike fitted with a 5 µm filter, with the latter showing lower cyclophosphamide concentrations, suggesting partial retention of the drug. No significant differences were found between the needle and filterless spike preparations. **Conclusions**: These results suggest that the choice of aspiration device influences the final drug concentration, potentially affecting therapeutic efficacy. Standardisation of preparation techniques and an awareness of device limitations are essential to ensure accurate chemotherapy dosing and patient safety.

## 1. Introduction

Cancer is a major global health crisis of the 21st century, impacting societies, public health and economies. It causes nearly 17% of all deaths and over 22% of noncommunicable disease (NCD)-related deaths. Among people aged 30–69 years, cancer accounts for 30% of premature deaths from NCDs and is a leading cause of death in most countries worldwide [1,2]. Chemotherapy has long been the mainstay of treatment for various cancers worldwide [3]. Chemotherapy involves the administration of cytotoxic and/or cytostatic drugs, which act by inhibiting cell proliferation and tumour growth, thereby preventing invasion and metastasis. Cytotoxic drugs affect cell replication (inhibition of the synthesis of new DNA strands and cell mitosis) by causing irreparable damage to DNA, leading to cell death. Chemotherapy can be administered as primary therapy for therapeutic effect or symptom control, in combination or not with other treatment strategies (e.g., monoclonal antibodies), or as a neoadjuvant or adjuvant therapy before or after surgery or radiotherapy [4]. Despite its widespread use and relevance in the context of cancer treatment, chemotherapy often causes significant side effects. Because these cytotoxic agents affect DNA and protein in both cancer and healthy cells, their therapeutic window is narrow. Treatment with these agents can lead to a range of toxic outcomes, including bone marrow suppression (myelosuppression), nausea and vomiting, various gastrointestinal problems, mucositis, hair loss (alopecia), sterility, infertility and infusion reactions. Immunosuppression further elevates the risk of infection [4]. Therefore, administration of the exact therapeutic dose is critical to obtaining a maximum therapeutic effect with minimum toxicity.

The most commonly used cytotoxic drugs in chemotherapy protocols are the alkylating agents, with cyclophosphamide being one of the most frequently prescribed [5]. Cyclophosphamide, classified as a nitrogen mustard alkylating agent, exerts its therapeutic effects by damaging DNA. This process is not dependent on a specific phase of the cell cycle. As a pro-drug, cyclophosphamide requires activation in the liver to form its active intermediaries, which disrupt protein synthesis by creating crosslinks between DNA and RNA [6]. Cyclophosphamide has many therapeutic indications and is used in the treatment of various types of cancer: breast, head and neck, lung, cervical, testicular and ovarian cancers, lymphomas (Hodgkin’s and non-Hodgkin’s lymphoma, lymphocytic lymphoma, small lymphocytic lymphoma, Burkitt’s lymphoma), leukaemia and multiple myelomas [7,8]. The use of cyclophosphamide raises significant concerns about bladder and reproductive toxicity. Studies and clinical trials have frequently reported side effects such as haemorrhagic cystitis, amenorrhoea, myelosuppression, alopecia and episodes of nausea and vomiting [9,10]. Cyclophosphamide treatment is tailored to each patient, considering factors such as their metabolism, potential drug interactions, body mass and age. Cyclophosphamide can be administered orally or intravenously. While oral formulations of cyclophosphamide are available, the intravenous route remains preferred in many protocols due to its complete bioavailability and need for controlled pharmacokinetics. Moreover, sustained-release formulations are not currently available, given that cyclophosphamide requires hepatic activation and exhibits a relatively short plasma half-life. Treatment guidelines provide appropriate dose regimens and indications based on specific treatment goals. In general, for patients without blood-related deficiencies and when used as the only oncolytic drug therapy, the recommended dose of intravenous cyclophosphamide is 40 to 50 mg/kg, administered in divided doses over 2 to 5 days [11]. Other intravenous regimens include 10 to 15 mg/kg every 7 to 10 days or 3 to 5 mg/kg twice weekly.

Because intravenous administration provides complete bioavailability, it is the most common route for delivering chemotherapy agents [4]. However, this route of administration requires handling and preparation of the pharmaceutical formulations to be administered to patients within the hospital units. Unlike oral formulations, which are generally ready for administration with little or no manipulation, cytotoxic drugs for intravenous administration are marketed as concentrated solutions or powders for reconstitution, which require dilution in appropriate solvents at a minimum. Needles are commonly used in these procedures, but the level of safety offered by these devices is insufficient for handling hazardous drugs. Therefore, several organisations, such as the International Society of Oncology Pharmacy Practitioners (ISOPP), the Association of Health-System Pharmacists (ASHP) and the National Institute for Occupational Safety and Health (NIOSH), advocate for the use of needle-free alternative systems whenever possible for the preparation of cytotoxic drugs, particularly closed system transfer devices (CSTDs) [12,13,14]. The promotion of safer handling by CSTDs compared to needle handling has been extensively described in previous studies [15,16,17,18,19,20,21]. Spikes are another alternative to needles. As with CSTDs, spikes minimise the risk of accidental needlesticks and help regulate pressure within the vial [22]. However, a key difference is that CSTDs, unlike standard spikes, also restrain hazardous aerosols, further reducing the risk of exposure [12]. Although the benefits associated with the use of CSTDs are more widely reported, studies have shown that there are countries, such as Portugal, where the use of spikes is much more common than the use of CSTDs [23]. This trend may be related to the lower cost and ease of use of spikes compared to CSTDs [22].

The aim of this study was to validate a high-performance liquid chromatography coupled to a diode-array detector (HPLC-DAD) analytical method for the detection and quantification of cyclophosphamide, which was further used to evaluate the performance of different aspiration devices—needles and spikes—on the final drug concentration after reconstitution.

## 2. Results

### 2.1. Chromatographic Analysis

**Figure 1:**
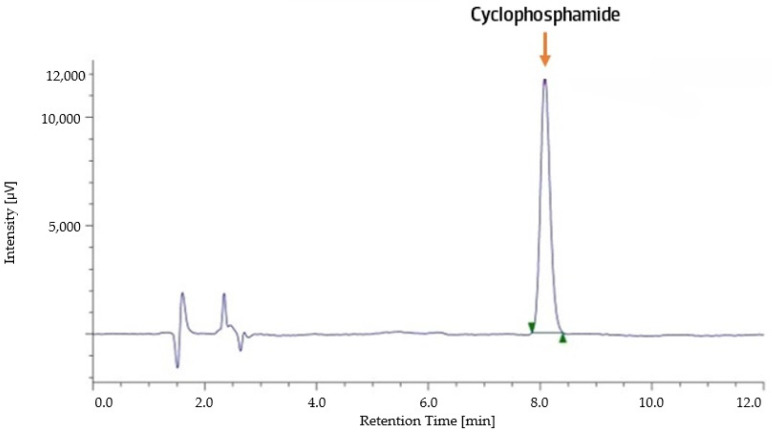
HPLC-DAD chromatogram of a 400 µg/mL cyclophosphamide standard solution.

Cyclophosphamide exhibited a retention time of 8.08 min (Figure 1).

### 2.2. Method Validation

#### 2.2.1. Linearity

The mean values and respective standard deviations of the chromatographic peak areas of six independent calibrators in triplicate are given in Table 1.

**Figure 2:**
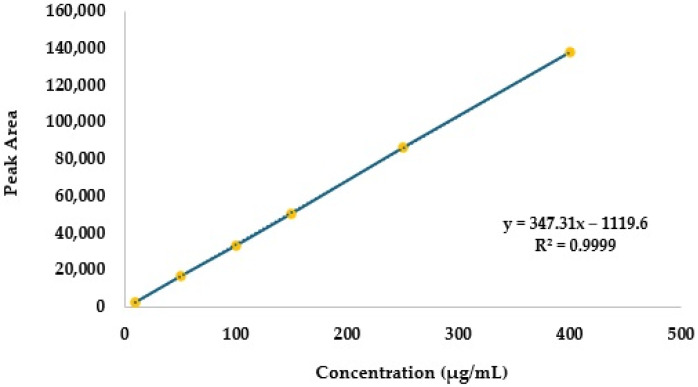
Cyclophosphamide calibration curve (10–400 µg/mL).

The calibration curve and the corresponding equation, which establishes the relationship between the equipment signal and the concentration of unknown cyclophosphamide samples, are depicted in Figure 2. An R^2^ value of 0.9999 was obtained, indicating excellent linearity.

The ANOVA test for linearity yielded a *p*-value lower than 0.001, confirming statistically significant linearity.

#### 2.2.2. Analytical Thresholds

A mean peak area of 150.1 ± 39.9 was achieved from the analysis of the blank injections. In this line, the limit of quantification (LOQ) and limit of detection (LOD) obtained were 1.15 µg/mL and 0.34 µg/mL, respectively, as determined based on the standard deviation (σ) of 39.9 and calibration curve slope (S) of 347.31.

#### 2.2.3. Specificity

**Figure 3:**
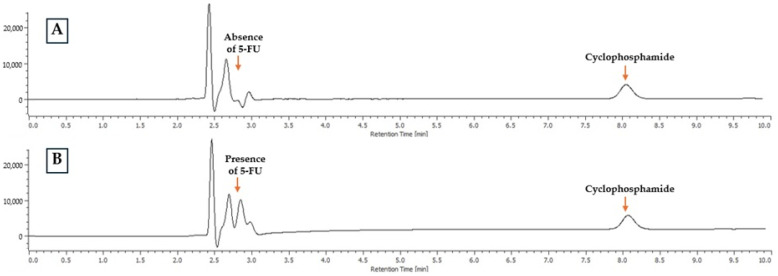
HPLC-DAD chromatograms of a 50 µg/mL cyclophosphamide standard solution (**A**) and a 50 µg/mL cyclophosphamide, paclitaxel and 5-FU standard solution (**B**).

As described above, 30 samples without cyclophosphamide were analysed in triplicate. The mean peak area obtained (150.1 ± 39.9) illustrates the stability of the baseline at the retention time characteristic of cyclophosphamide. When cyclophosphamide samples spiked with 5-fluorouracil (5-FU) and paclitaxel (50 µg/mL) were injected, a sample area of 16,034 ± 222 was observed (Figure 3). This value is similar (−2.5%) to the sample area of the chromatographic peak of cyclophosphamide injected alone (16,442 ± 238) (Figure 3A).

#### 2.2.4. Precision and Accuracy

For repeatability, the coefficient of variation (CV) ranged from 1.51% to 2.66%, while for intermediate precision, the CV ranged from 0.22% to 4.59%. Accuracy ranged from 89.8% to 99.4% for the three concentrations evaluated (Table 2).

#### 2.2.5. Robustness

A pH reduction of 0.2 units had no effect on the cyclophosphamide peak area or retention time, maintaining the same profile as the optimised method. Conversely, increasing the flow rate by 0.2 mL/min resulted in a decrease of 0.43 min in retention time (Table 3).

#### 2.2.6. Stability

The results of the assessment of cyclophosphamide stability are depicted in Table 4.

### 2.3. Quality Control of Cyclophosphamide Solutions Aspirated with Different Devices

In the present study, chromatographic peaks and corresponding cyclophosphamide peak areas were evaluated after aspirating reconstituted solutions from the original vial using distinct devices: (i) a needle, (ii) spike A, and (iii) spike B. In 17 of the reconstituted vials, aspiration was performed first with a needle, followed by aspiration with spike A. In 14 additional vials, aspiration was performed first with a needle and then with spike B. All samples had a theoretical concentration of 200 µg/mL. The peak area values obtained from the analysis were converted to cyclophosphamide concentration using the calibration curve (*y* = 347.31*x* − 1119.6) depicted in Figure 2 (Table 5 and Table 6).

**Figure 4:**
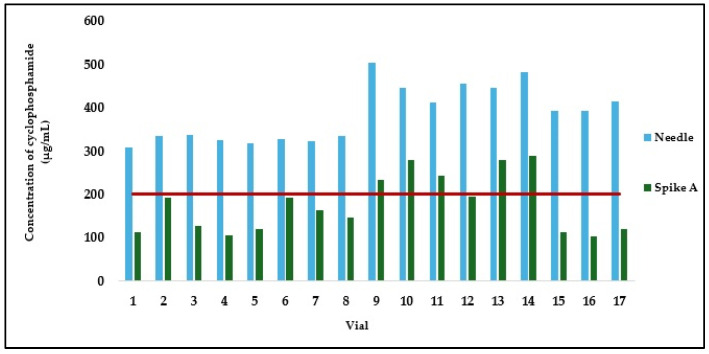
Cyclophosphamide concentration in samples taken from the same vial by operators A and B, using either a needle or spike A. The red line indicates theoretical concentration (200 µg/mL).

Figure 4 presents a graphical comparison of the concentrations of samples withdrawn using a needle or spike A. In the group of samples handled by operators A and B, the use of a needle resulted in a mean cyclophosphamide concentration of 385.51 ± 64.18 µg/mL, while spike A yielded 177.55 ± 66.49 µg/mL, corresponding to a statistically significant mean difference of 207.96 ± 52.40 µg/mL (*p* < 0.001; Table 7). The coefficient of variation was 16.65% for the needle and 37.45% for spike A, indicating high variability, particularly in the latter. Notably, the needle nearly doubled the mean concentration obtained with spike A. Individual concentrations reached values as high as 503.20 µg/mL (vial 9), well above the theoretical value of 200 µg/mL, demonstrating a marked deviation in the preparations. Even the lowest concentration obtained with a needle, 308.84 µg/mL (vial 1), was already substantially higher than the theoretical concentration. In all 17 paired observations, the concentrations of the samples aspirated with a needle were consistently higher than those obtained with spike A. Furthermore, spike A showed considerable variability in performance, with concentrations ranging from 105.38 µg/mL (vial 4) to 289.13 µg/mL (vial 14), the latter being 2.7 times higher than the former. In some cases, the difference in concentration between the two devices for the same sample was almost threefold, as seen in vial 17 (415.37 µg/mL with needle vs. 119.93 µg/mL with spike A).

**Figure 5:**
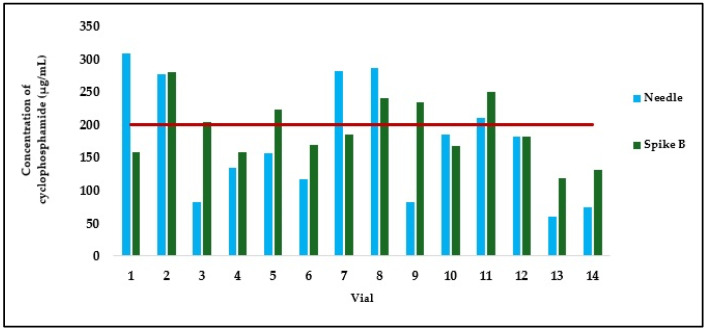
Cyclophosphamide concentration in samples taken from the same vial by operators C and D using either a needle or spike B. The red line indicates the theoretical concentration (200 µg/mL).

In the group of samples handled by operators C and D, the mean cyclophosphamide concentrations obtained with the needle and spike B were 174.27 ± 81.45 µg/mL and 193.22 ± 48.05 µg/mL, respectively. These averages were closer to each other compared to those observed with spike A. However, a marked variability was again noted, particularly with the needle (CV 46.74% for the needle versus 24.87% for spike B). Although individual concentrations with the needle reached as high as 308.46 µg/mL (vial 18), the mean remained closer to the theoretical value of 200 µg/mL, likely due to compensation by markedly lower values, such as 60.56 µg/mL in vial 30, which represents approximately one-quarter of the expected concentration. Despite the fact that the mean value obtained with spike B (193.22 µg/mL) was the closest to the theoretical target among all devices tested, the observed concentrations still ranged widely, from 118.92 µg/mL (vial 30) to 280.77 µg/mL (vial 19). Furthermore, high intra-sample variability was also observed in this group, with differences in concentration between the needle and spike B reaching up to 151.72 µg/mL (vial 26), once again highlighting the substantial impact of the aspiration method on the final drug concentration. Even so, by comparing the concentrations obtained by aspiration using a needle and spike B, no clear or consistent trend was observed towards higher or lower concentrations across all preparations (*p* > 0.5, Wilcoxon test) (Figure 5).

## 3. Discussion

Cyclophosphamide is a widely used cytotoxic and immunosuppressive drug, integral to regimens in oncology (e.g., breast cancer, lymphomas), autoimmune diseases (e.g., lupus nephritis, vasculitis), and bone marrow transplant conditioning. Herein, we validated an HPLC-DAD method for the quantification of cyclophosphamide, which was subsequently used to evaluate the effect of aspiration devices on the final concentration of the drug in solutions intended for administration to patients undergoing cyclophosphamide treatment regimens. During the adaptation of the method previously described by Viegas et al. [24], injection volumes of 100 µL (as described in the original study) and 10 µL were tested. The lower injection volume resulted in a better chromatographic peak profile, with reduced peak tailing, facilitating a more accurate peak integration and contributing to greater sensitivity and resolution. In addition, a lower injection volume minimised baseline instability, which improved subsequent analyses and enhanced the reproducibility of the method [25]. Moreover, it reduced sample consumption, a particularly valuable advantage when working with limited sample amounts. The observed retention time of 8.08 min aligns with the values reported in previous studies that also employed HPLC-UV analysis [26,27].

Linearity can be assessed by analysing the graphical representation of the calibration curve and is usually considered valid when the correlation coefficient (R^2^) is ≥0.995. In this study, a visual inspection of the calibration curve and a correlation coefficient of 0.9999 suggest a strong linear relationship between the two variables. A *p*-value < 0.01 (ANOVA test) further confirms the statistical significance of this relationship. Additionally, the differences between actual concentration and back-calculated concentrations after model development provide further evidence of its validity [28,29]. In the present study, only slight deviations were observed in the back-calculated concentration values, with differences ranging from −1% and 4.8%, further supporting a linear relationship between the concentration and equipment signal.

The obtained LOQ (4.03 µg/mL) and LOD (1.21 µg/mL) values are slightly lower than those previously reported, 5.40 µg/mL and 1.78 µg/mL, respectively, in a study using HPLC-DAD for cyclophosphamide detection [24]. Even so, studies employing liquid chromatography coupled with tandem mass spectrometry (LC-MS/MS) have reported significantly lower values, such as 0.1 ng/mL for LOQ and 0.005 ng/mL for LOD [30]. Also, ultra-performance liquid chromatography coupled with tandem mass spectrometry (UPLC-MS/MS) has achieved LOQ and LOD values of 3.5 ng/mL and 1 ng/mL, respectively [31]. Additionally, gas chromatography coupled with mass spectrometry (GC-MS) has been used to develop analytical methods with LOQ values as low as 0.0077 ng/mL and LOD values of 0.00023 ng/mL [32]. These findings are not entirely surprising, considering that GC-MS, LC-MS/MS and UPLC-MS/MS are inherently more sensitive methodologies than HPLC-DAD. However, sensitivity in the range achieved by LC-MS/MS, UPLC-MS/MS or GC-MS is, in this case, unnecessary. The reconstituted cyclophosphamide solution has a concentration of 20 mg/mL, and after dilution, a final concentration of at least 2 mg/kg in 250 mL 0.9% NaCl is obtained (which corresponds to a final concentration of 0.56 mg/mL of cyclophosphamide in a 70 kg patient) [11]. In fact, such high sensitivity may even be counterproductive, as further dilutions would be required to fit the measured concentration within the linear range of the methods. Additionally, the goal of this study was to establish a method suitable for the routine quality control of chemotherapy preparations in most central hospitals. Advanced techniques such as LC-MS/MS, UPLC-MS/MS and GC-MS are unlikely to be widely available as HPLC-DAD or HPLC/UV, making the latter a more practical choice for hospital laboratories. Nevertheless, future studies could explore the application of more sensitive techniques, such as LC-MS/MS, for trace analysis in biological matrices like blood, thereby expanding the scope of cyclophosphamide therapeutic monitoring and ensuring patient safety.

Another potential disadvantage of HPLC-DAD technology is the lower discriminatory power when analysing samples prone to contamination or containing multiple foreign compounds. Unlike mass spectrometry-based techniques, HPLC-DAD may lead to the misinterpretation of findings in the presence of potential interferents. In this sense, it is important to consider that the solutions analysed herein are not expected to contain multiple contaminants that could impact the HPLC-DAD baseline signal. The cyclophosphamide samples are composed solely of a reconstitute solvent (usually water for injection or 0.9% NaCl) and a diluent solvent (typically 0.9% NaCl). While it is possible that pharmacy professionals may inadvertently introduce another drug into the injection bag instead of cyclophosphamide, the most commonly handled drugs in chemotherapy preparation units—paclitaxel and 5-FU [23]—were analysed together with cyclophosphamide in HPLC-DAD injections. The results showed no co-elution with cyclophosphamide: paclitaxel was not detected, eventually due to a higher affinity with the stationary phase and, as such, longer retention time, and 5-FU eluted at the beginning of the chromatographic run, coinciding with the injection peak (Figure 3). The similarity of cyclophosphamide peak areas when analysed alone or simultaneously with 5-FU and paclitaxel confirms that these drugs do not interfere with cyclophosphamide detection. This means that if an incorrect preparation occurred (e.g., accidental substitution with 5-FU or paclitaxel), HPLC-DAD would not detect any peak at the retention time characteristic of cyclophosphamide, ensuring the method’s specificity. Nonetheless, it should be noted that only two potential interferents were evaluated in this study. Although 5-FU and paclitaxel were selected due to their high prevalence in chemotherapy preparations, the inclusion of a broader panel of potential interferents would provide a more comprehensive evaluation of method specificity. Future studies should expand the range of tested compounds to further validate the method under more complex clinical scenarios.

Since the repeatability and intermediate precision of the method yielded values below the recommended 15% limit, it can be concluded that the method is reproducible [33,34]. The results obtained during the accuracy evaluation demonstrated a close agreement between the experimentally obtained values and the reference values [33]. Compliance with the reference thresholds was also confirmed, which, in this case, are set between 85% and 115% according to the International Council for Harmonisation of Technical Requirements for Pharmaceuticals for Human Use (ICH) guidelines [34].

The ICH guidelines recommend assessing the HPLC method’s robustness by varying the mobile phase conditions [35]. In this study, altering the mobile phase pH or flow rate did not significantly impact cyclophosphamide peak intensity. Increasing the flow rate resulted in only a minor shift in retention time, further supporting the robustness of the method. Although a higher flow rate allowed for faster elution, it also led to greater solvent consumption per unit of time. For this reason, the authors opted to maintain the conditions described elsewhere [24,36].

A compound is considered stable over a specific period if its concentration does not vary by more than 15% [37]. Cyclophosphamide solutions at three concentration levels (35, 60 and 185 µg/mL) were found to remain stable after 24 h at room temperature, in the refrigerator, and in the freezer, as the observed variations were all below the 15% threshold relative to the initial concentration. These findings suggest that cyclophosphamide remains stable in solution for a time sufficient for its analysis, provided that the hospital unit performing the preparation has access to an HPLC-DAD system. Additionally, the Endoxan^®^ summary of product characteristics (SmPC) states that once dissolved in the diluent, the drug remains stable for 24 to 48 h and that after reconstitution, the drug must be used within 24 h. Since this study focused on the evaluation of the reconstituted solutions, the stability evaluation was limited to 24 h, which is consistent with the SmPC and reflects standard hospital preparation and administration practices. In future studies, it would be interesting to explore extended stability under various storage conditions to ensure broader applicability of the method.

**Figure 6:**
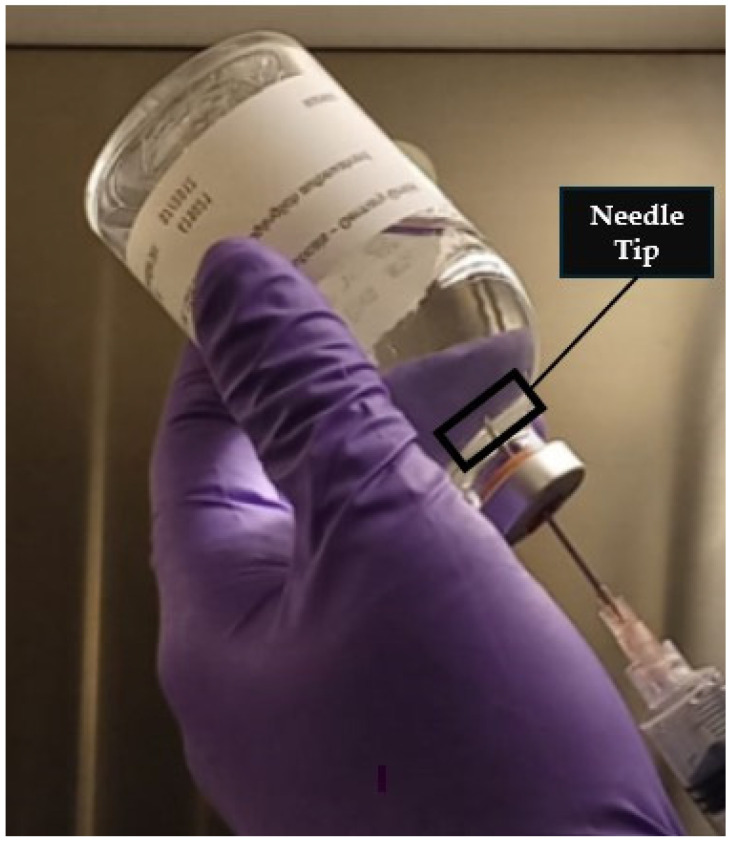
Withdrawal of cyclophosphamide solution from the vial, with the needle tip positioned near the elastomer (highlighted).

The results from the analysis of real preparations indicate high variability in the concentrations of cyclophosphamide withdrawn from the vial, regardless of the professional performing the preparation or the piercing device used, with coefficients of variation equal to or exceeding 16.65%. These findings suggest a highly heterogeneous distribution of the powder in the solvent, which can have negative consequences when the entire vial is not used in a single preparation. This variability may compromise compliance with prescribed therapeutic regimens, as lower-than-prescribed concentrations could reduce treatment efficacy, while higher-than-prescribed concentrations may place the patient at increased risk of toxic effects. A careful analysis of the results obtained using needles by professionals 1 and 2 for the first 17 vials (mean concentration of 385.51 ± 64.18 µg/mL) and by professionals 3 and 4 for vials 18 to 31 (mean concentration of 174.27 ± 81.45 µg/mL) reveals significant differences between these two sets of manipulations. This finding reinforces the idea that proper dilution of cyclophosphamide is a key factor in ensuring the correct dose is administered to the patient. Although professionals were instructed to perform the procedures as they routinely do and reported that they “shook the solution until no visible particles remained in the vial”, the actual shaking technique may have varied significantly between professionals. In preparations 1–17, a consistently higher-than-theoretical cyclophosphamide concentration was observed. This may be attributed to the deposition of undissolved cyclophosphamide powder near the elastomer. When inverting the vial for aspiration, the needle must be submerged in the liquid, which often leads professionals to position the needle tip close to the elastomer. Simple agitation may have resuspended the deposited powder into the solution, potentially explaining the differences and variability observed in preparations 18–31 (Figure 6).

The variation in the results between professionals is substantial and highlights the need to emphasise proper reconstitution techniques and ensure the homogeneous distribution of cyclophosphamide in solution. The SmPC of cyclophosphamide (Endoxan^®^, Bayer) recommends “vigorous shaking” until complete dissolution during drug reconstitution. However, this guidance is generic and lacks clear, standardised instructions on how the preparation should be carried out. Future studies could help to identify optimal reconstitution strategies, including the impact of temperature on solubility, the minimum shaking duration required and the specific shaking movements that promote better dilutions. Training programs for healthcare professionals should incorporate education on proper reconstitution techniques to minimise concentration variability. Observational studies conducted in European countries have already highlighted insufficient training among professionals handling cytotoxic drugs [23,38].

A maximum variation of 5% between the prepared dose and the prescribed dose is generally considered acceptable. It is expected that such dose adjustments in biological and cytotoxic agents do not compromise treatment safety or efficacy [39]. This dose adjustment strategy is also recognised as a measure to reduce waste and healthcare costs [39,40]. In the present study, only three preparations remained within the acceptable 5% deviation from the expected theoretical dose. The observed dose variations, when compared to the expected theoretical dose, were between 30.3% (vial 30) and 251.6% (vial 9) for preparations made using a needle, between 52.1% (vial 16) and 144.6% (vial 14) for preparations made using spike A; and between 59.5% (vial 30) and 140.4% (vial 19), for preparations made using spike B. Given the narrow therapeutic index of cytotoxic agents like cyclophosphamide, these results reveal a critical issue with potentially serious clinical consequences. Deviations from the prescribed dose as large as those observed in this study can significantly compromise both the safety and efficacy of cyclophosphamide therapy. Overdosage, as evidenced by concentrations exceeding the theoretical dose by more than 200% in some cases, may substantially increase the risk of severe toxicities, including myelosuppression, immunosuppression and organ toxicity, all of which can be life-threatening. This concern is supported by clinical evidence from a study involving Kenyan children with endemic Burkitt lymphoma, where patients inadvertently overdosed by more than 115% of the correct cyclophosphamide dose had an increased risk of mortality (adjusted Hazard Ratio 1.43, 95% CI: 0.84–2.43) compared to those receiving accurate dosing [41]. Conversely, underdosing may lead to poorer clinical outcomes across its uses as a result of subtherapeutic drug exposure that potentially leads to reduced treatment efficacy, higher risk of disease progression or relapse, and, in some cases, contributes to the development of drug resistance, all of which carry severe health, economic and social impacts. Accordingly, in oncology, a reduction in chemotherapy dose or intensity often translates to reduced tumour kill, lower remission rates and a higher probability of recurrence. Patients receiving substantially less than the recommended chemotherapy have been shown to have worse survival in diseases like breast cancer and lymphoma [42,43]. Furthermore, subtherapeutic dosing facilitates the survival of drug-resistant cancer cell clones, potentially making the disease more refractory. As some studies noted, failing to completely eradicate cancer can accelerate the emergence of a resistant tumour population [44]. In autoimmune diseases, underdosing cyclophosphamide means insufficient immunosuppression. Clinical studies in lupus and vasculitis demonstrate that while careful dose minimisation is possible, going below the effective dose thresholds leads to worse remission rates and more relapses [45,46]. Also, in bone marrow transplantation, underdosing can defeat the purpose of the procedure, causing graft rejection, early relapse of malignancy or inadequate graft-versus-host disease prophylaxis [47,48]. To avoid these consequences, expert guidelines and practitioners generally advise against arbitrary dose reductions. For example, maintaining at least 85% of the planned dose intensity in curative chemotherapy is recommended as quality care [49,50]. No significant differences were, however, found in a retrospective study involving cyclophosphamide underdosing in patients with diffuse large B-cell lymphoma [51]. Nevertheless, the sample size was limited, the exact dose reduction per patient was unknown and the extent of underdosing was relatively small (estimated 3–20%), potentially limiting the study’s ability to detect clinically relevant differences. To the best of our knowledge, no other studies have evaluated the clinical impact of underdosing at the high levels observed in our study, leaving the potential repercussions of such significant deviations from the prescribed dose largely unknown. Yet, all available evidence, from clinical trials to real-world studies, highlights the consequences of dosing inaccuracies and further reinforces the critical importance of ensuring precise chemotherapy preparation and administration.

The reconstituted vials were first sampled using a needle and then immediately with a spike. This approach allowed for paired observations, enabling the assessment of the statistical significance of the differences. The ability to analyse the contents of the same vial using two different methods reduces the potential for bias and interference from other variables that could otherwise explain the differences. On average, preparations made with a needle had a 2.17-fold higher concentration than those prepared using a spike equipped with a 5 μm liquid/particle filter and a 0.2 μm ventilation filter. In all 17 paired observations, samples withdrawn using a needle consistently resulted in higher cyclophosphamide concentrations (*p* < 0.001). This suggests that the aspiration device plays a critical role in chemotherapy preparation and directly impacts the quality of the final drug product. A possible explanation for these results is a phenomenon of cyclophosphamide aggregation or agglomeration after reconstitution. As a result, some drug retention may occur within the spike filter, possibly due to partial retention or obstruction.

## 4. Materials and Methods

### 4.1. Reagents

All chemicals and reagents were of analytical grade or the highest commercial quality. Acetonitrile (gradient grade, for HPLC, ≥99.9%; 41.05 g/mol) was purchased from Fisher Scientific^®^ (Loughborough, UK), and methanol (gradient grade, for HPLC, ≥99.9%; 32.04 g/mol) from VWR Avantor^®^ (Fontenay-sous-Bois, Paris, France). Cyclophosphamide monohydrate (≥97% purity, 279.10 g/mol), 5-FU (>98% purity, 279.10 g/mol), and paclitaxel (≥95% purity, 853.91 g/mol) were acquired from Sigma-Aldrich^®^ (St. Louis, MO, USA).

### 4.2. Calibrators and Quality Control Samples

Stock solutions of cyclophosphamide at 10 mg/mL, 5-FU at 400 µg/mL and paclitaxel at 400 µg/mL were prepared in methanol, acetonitrile and water (13:19:68), vortex mixed, and stored at 4 °C. Cyclophosphamide calibrators (10, 20, 50, 90, 100, 150, 250 and 400 µg/mL) were further prepared by diluting the stock solution. After preparation, all solutions were filtered with a 0.2 µm polytetrafluoroethylene filter, and chromatographic analysis was conveniently performed.

### 4.3. HPLC-DAD Analytical Settings

Reverse-phase chromatography using a non-polar stationary phase and a polar mobile phase was performed on a JASCO^®^ HPLC-DAD system with a C-18 HypersilGOLD^®^ column (15 mm × 4.6 mm × 5 µm) from Fisher Scientific^®^ (Loughborough, UK). The HPLC-DAD was equipped with an MD-4010 DAD, an AS-4050 autosampler, a PU-4080-LG pump and a CO-4061 oven. The equipment was connected to a computer running ChromNav^®^ version 2.0 software for data acquisition and analysis.

The chromatographic method was adapted from Viegas et al. [24]. Briefly, elution was performed in the isocratic mode at a flow rate of 0.8 mL/min using a mobile phase composed of acetonitrile, methanol and water (19:13:68). A different injection volume was used compared to the reference study: 10 µL instead of 100 µL. The chromatographic run time was set to 12 min, and the detection wavelength was 205 nm, as described elsewhere [30].

To prepare the mobile phase, water was filtered through a 0.22 µm cellulose acetate membrane filter using a vacuum pump. All components of the mobile phase were then degassed by ultrasonication (Bandelin Sonorex^®^, Sigma-Aldrich, St. Louis, MO, USA) for 30 min at room temperature.

### 4.4. Method Validation and Acceptance Criteria

Following the recommendations of the ICH [35], the following parameters were evaluated for validation purposes: linearity, analytical thresholds, i.e., LOD and LOQ, specificity, precision (intermediate precision and repeatability), accuracy and robustness. Stability was also evaluated.

#### 4.4.1. Linearity

The calibration curve was plotted using standard solutions at six concentration levels: 10, 50, 100, 150, 250 and 400 µg/mL, in accordance with the ICH requirements (i.e., a minimum of five calibrators) [33,52]. Each standard (calibrator) was injected in triplicate, and the process was repeated six times, resulting in six independent calibration curves. The results were then combined to produce a final calibration curve based on the average of all analyses. Linearity was assessed by visual inspection of the calibration curve, calculation of the correlation coefficient and agreement after the back-calculation.

#### 4.4.2. Limits of Detection and Quantification

The LOD and LOQ were determined based on the standard deviation (σ) of 30 blanks consisting of water for injections (the main component of the mobile phase and the solvent used for reconstitution and dilution of commercially available cyclophosphamide powder for chemotherapy). These blanks were analysed in triplicate, and the values, together with the slope (S) of the calibration curve, were used to calculate the LOD and LOQ using the following equations:

LOD = 3.3 × σ/S,(1)

LOQ = 10 × σ/S,(2)

#### 4.4.3. Specificity

The absence of an interference method was used to assess specificity [35]. Specificity, defined as the ability to unequivocally assess the analyte in the presence of components that may be expected to be present, was evaluated by spiking three vials with 5-FU, paclitaxel and cyclophosphamide, each at a final concentration of 50 µg/mL. Paclitaxel and 5-FU were selected as the potential interferents since, along with cyclophosphamide, they are the most commonly handled cytotoxic drugs and, therefore, most prone to inadvertent transfer [23]. In addition, the previously described analysis of the 30 blanks in triplicate was used to confirm the absence of interferents in samples not containing cyclophosphamide.

#### 4.4.4. Precision

To determine precision, the CV or relative standard deviation (RSD) was calculated using the standard deviation (σ) and the mean (X) of the cyclophosphamide peak areas, as shown in Equation (3):

RSD (%) = σ/*X* × 100 (3)

To determine repeatability (also known as intra-day precision), five samples of three cyclophosphamide concentrations (20, 50 and 150 µg/mL) were analysed in triplicate by the same operator on the same day. For the determination of intermediate precision, the same concentrations were analysed in triplicate on three different days. These procedures meet the requirements for the determination of repeatability and intermediate precision [33,35].

#### 4.4.5. Accuracy

Three different cyclophosphamide concentrations (20, 50 and 150 µg/mL) were used to assess the accuracy of the method. The expected concentration (C_e_) was back-calculated from the calibration curve and then compared with the nominal or theoretical concentration (C_t_) and assessed by independently preparing and analysing three replicates of each calibrator (Equation (4)).

Accuracy = (C_e_/C_t_) × 100 (4)

#### 4.4.6. Robustness

Robustness was evaluated based on minor changes in the mobile phase pH (0.2 decrease) and flow rate (0.2 mL/min increase), as per the ICH guidelines [35], with resulting differences in the peak area and retention time compared to the optimised method. Acetic acid (60.05 g/mol), purchased from VWR^®^ (Fontenay-sous-Bois, Paris, France), was used to promote acidification of the pH. The robustness evaluation was performed at 50 µg/mL cyclophosphamide in triplicate.

#### 4.4.7. Stability

For the determination of stability, cyclophosphamide samples at different concentrations of the linear interval (30, 60 and 185 µg/mL) were prepared and analysed in triplicate immediately and 24 h later after storage under different temperature conditions: room temperature (approximately 25 °C), refrigeration (approximately 4 °C) and freezing (approximately −20 °C). Stability was calculated using Equation (5), comparing the mean area of the peaks for each concentration at the start of the stability study (Ā_0_) and at the end of the stability study (Ā_24_) (after 24 h).

Stability_i_ = (Ā_24_)/(Ā_0_) × 100 (5)

### 4.5. Proof of Applicability

**Figure 7:**
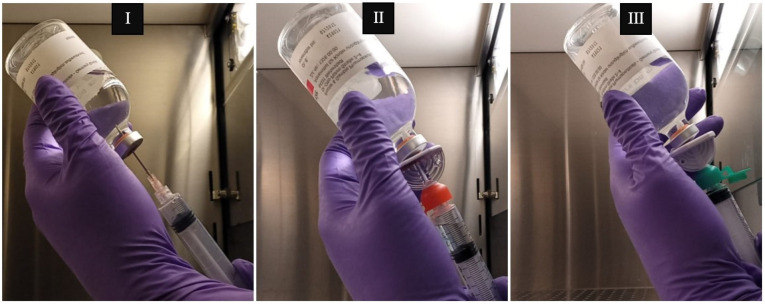
Representative figures of cyclophosphamide solution withdrawing using an 18G needle (**I**), spike A (**II**) and spike B (**III**) as puncture devices.

For quality control, cyclophosphamide solutions at 20 mg/mL were prepared and sampled using different aspiration devices. Accordingly, commercial vials of 1000 mg of cyclophosphamide (Endoxan^®^, Baxter, Deerfield, IL, USA) were reconstituted with 50 mL of water for injection using syringes with an absolute capacity of 60 mL (nominal capacity of 50 mL; B. Braun—Omniflex^®^, Toronto, ON, Canada) coupled with hypodermic needles (1.20 × 40 mm/18 G × 1 1/2″; Medoject^®^, Chirana T. Injecta, Stará Turá, Slovakia). After reconstitution, 1 mL of the solution was withdrawn from the vial using syringes of a 3 mL absolute capacity (nominal capacity of 2 mL; B.Braun Injekt SOLO^®^, B. Braun Medical Lda., Lisbon, Portugal), either coupled with a hypodermic needle (1.20 × 40 mm/18 G × 1 1/2″; Medoject^®^), spike A (spike marketed as adequate for handling cytotoxic drugs and equipped with a 5 μm liquid/particle filter and a 0.2 μm ventilation filter) or spike B (equipped with a 0.45 μm ventilation filter), and transferred into a new vial. Then, a volume of 0.1 mL of the collected sample was always transferred into a centrifuge tube containing 9.9 mL of methanol, acetonitrile and water (13:19:68), resulting in a solution with a theoretical concentration of 200 µg/mL. This dilution ensured that the samples had a theoretical concentration within the linear range of the validated analytical model. A total of 31 vials were reconstituted. Then, a first collection of 1 mL of the reconstituted solution was made from each vial using the syringe coupled to a needle. Then, from the same vials, a 1 mL collection was performed using a syringe coupled with either spike A (vials 1 to 17) or spike B (vials 18 to 31) (Figure 7).

All the reconstitution and aspiration procedures were carried out by trained and experienced senior pharmacy technicians in accordance with the recommendations of the product package leaflet and were subsequently double-checked by a second experienced senior pharmacy technician. In accordance with the service schedule of the hospital unit where the sample collection was performed, vials 1 to 17 were processed by professional A with double-checking by professional B; and vials 18 to 31 were processed by professional C with double-checking by professional D. Chromatographic analysis was then performed under the same conditions as above.

### 4.6. Statistical Analysis

All statistical analyses were performed using IBM^®^ SPSS Statistics software (version 29.0.1.0). The normality of the data was assessed using the Shapiro–Wilk test. Statistical comparisons between groups were performed using the Student’s paired *t*-test (when the data followed a normal distribution) or the Wilcoxon test (when the data did not follow normality) at a significance level of 0.05. In addition, the statistical significance of the method’s linearity was assessed using ANOVA.

## 5. Conclusions

This study highlights the significant impact of aspiration devices and handling techniques on the final concentration of cyclophosphamide preparations for chemotherapy. The results demonstrate that samples aspirated using spikes equipped with a 5 μm liquid/particle filter and a 0.2 μm ventilation filter consistently showed lower cyclophosphamide concentrations compared to those aspirated with a needle, suggesting that the piercing device plays a critical role in ensuring dose accuracy. While the exact mechanism behind this systematic difference remains unclear, potential explanations include drug retention within the filter or variations in the aspiration process. Additionally, a substantial variability in final concentrations was observed among different operators, likely resulting from differences in homogenization techniques and aspiration procedures.

The HPLC-DAD method validated herein proved to be accurate, precise, robust and suitable for quality control in hospital settings. Although the method was validated in accordance with the ICH guidelines, future studies should expand the validation to include a broader range of potential interferents, stability testing over extended periods, and robustness evaluations under various operational scenarios (e.g., variations in the column temperature, minor changes in the mobile phase composition or performance comparisons across different operators) to further ensure the method’s applicability to real-world clinical environments. Regular application of such analytical methods is essential to monitor the accuracy of cytotoxic drug preparations, detect deviations early and ensure that patients receive the correct dose. This is particularly relevant given the narrow therapeutic index of many anticancer agents and the potentially serious consequences of under- or overdosing. Given that chemotherapy efficacy and safety rely on the precise administration of the prescribed dose, these findings emphasise the need for the strict standardisation of preparation techniques and enhanced training for healthcare professionals involved in cytotoxic drug handling. Future studies should explore optimal reconstitution strategies, including shaking techniques, temperature effects and standardised agitation protocols, to minimise variability and ensure safe and effective chemotherapy administration.

## Figures and Tables

**Table 1:** Concentrations and corresponding mean peak areas of the calibrators used to plot the cyclophosphamide calibration curve.

Concentration (µg/mL)	Mean Peak Area	Standard Deviation	Relative Standard Deviation (%)	Back-Calculated Concentration (µg/mL)	Difference (%)
10	2506	172	6.86	10.48	4.8
50	16,442	238	1.45	50.75	1.5
100	33,125	356	1.07	98.97	−1.0
150	50,648	312	0.62	149.61	−0.3
250	86,372	232	0.27	252.85	1.1
400	137,604	237	0.17	400.91	0.2

**Table 2:** Evaluation of precision (repeatability and intermediate precision) and accuracy at three concentration levels of cyclophosphamide.

Concentration (µg/mL)	Repeatability		Intermediate Precision		Accuracy		
	Mean Area ± Standard Deviation	Relative Standard Deviation (%)	Mean Area ± Standard Deviation	Relative Standard Deviation (%)	Mean Area	Back-calculated Concentration (µg/mL)	Accuracy (%)
20	5220 ± 139	2.66	5119 ± 235	4.59	5118 ± 234	17.96	89.8
50	16,107 ± 280	1.74	16,442 ± 380	2.31	16,002 ± 335	49.30	98.6
150	51,769 ± 781	1.51	51,648 ± 115	0.22	50,648 ± 481	149.05	99.4

**Table 3:** Analytical data for robustness evaluation with respect to pH and flow rate parameters for the analysis of cyclophosphamide in triplicate (50 µg/mL).

Parameter	Parameter Setting	Peak Area (Mean ± Standard Deviation)	% of Peak Area in Relation to Optimised Method	Retention Time (Minutes)	Difference of Retention Time in Relation to Optimised Method (Minutes)
pH	6.6 *	15,883 ± 137	-	8.08	-
	6.4	15,660 ± 207	98.6	8.08	0.0
Flow rate	0.8 mL/min *	15,883 ± 137	-	8.08	-
	1.0 mL/min	15,152 ± 208	95.4	7.65	−0.43

* Optimised method.

**Table 4:** Peak area and stability of cyclophosphamide solutions at 30, 60 or 185 µg/mL assessed immediately after preparation (T_0_) and after 24 h storage (T_24_) at room temperature (25 °C), under refrigeration (4 °C) or freezing conditions (−80 °C).

	T_0_	T_24_					
Concentration (µg/mL)		Room Temperature (≈25 °C)		Refrigerator (≈4 °C)		Freezer (≈−20 °C)	
	Mean Peak Area	Mean Peak Area	Stability (%)	Mean Peak Area	Stability (%)	Mean Peak Area	Stability (%)
30	7525	7348	97.65	7526	100.01	7762	103.16
60	20,237	20,769	102.63	22,065	109.04	20,444	101.03
185	63,649	63,077	99.10	64,356	101.11	60,118	94.45

**Table 5:** Cyclophosphamide concentrations after withdrawal of the reconstituted solution from the vial using a needle and, subsequently, spike A, followed by a 1:100 dilution, as performed by operators A and B.

Vial Number	Concentration of Solution Withdrawn with Needle (µg/mL) *	Concentration of Solution Withdrawn with Spike A (µg/mL) *	Difference Between Withdrawal with Needle and Vial
1	308.84	112.23	196.61
2	333.78	193.11	140.67
3	336.66	128.23	208.43
4	326.06	105.38	220.68
5	318.16	120.23	197.93
6	327.36	192.44	134.92
7	323.46	164.44	159.02
8	334.37	146.87	187.50
9	503.20	234.39	268.81
10	445.76	278.91	166.85
11	412.45	243.70	168.75
12	454.12	194.77	259.35
13	446.24	278.91	167.33
14	482.78	289.13	193.65
15	393.13	111.56	281.57
16	391.87	104.14	287.73
17	415.37	119.93	295.44
Mean	385.51	177.55	207.96 (*p* < 0.001)
Standard Deviation	64.18	66.49	52.40
Coefficient of Variation (%)	16.65	37.45	25.20

* All samples had a theoretical concentration of 200 µg/mL.

**Table 6:** Cyclophosphamide concentrations after withdrawal of the reconstituted solution from the vial using a needle and, subsequently, spike B, as performed by operators C and D.

Vial Number	Concentration of Solution Withdrawn with Needle (µg/mL) *	Concentration of Solution Withdrawn with Spike B (µg/mL) *	Difference Between Withdrawing with Needle and Vial
18	308.46	157.93	150.53
19	277.13	280.77	−3.64
20	82.52	204.18	−121.66
21	134.88	158.19	−23.31
22	156.21	223.05	−66.84
23	117.23	169.70	−52.47
24	281.35	185.02	96.33
25	286.3	240.60	45.7
26	82.63	234.35	−151.72
27	185.15	167.70	17.45
28	210.80	250.33	−39.53
29	182.29	182.74	−0.45
30	60.56	118.92	−58.36
31	74.23	131.59	−57.36
Mean	174.27	193.22	−18.95
Standard Deviation	81.45	48.05	65.97
Coefficient of Variation (%)	46.74	24.87	−348.09

* All samples had a theoretical concentration of 200 µg/mL.

**Table 7:** Paired *t*-test analysis of the difference in cyclophosphamide concentrations obtained by aspiration with a needle or with spike A.

Paired Samples Test							
Paired Differences					t	dF	Sig. (2-tailed)
Mean	Standard Deviation	Standard. Error Mean	95% Confidence Interval of the Difference				
			Lower	Upper			
207.96	52.40	12.71	181.01	234.90	−16.36	16	<0.001

## Data Availability

The original contributions presented in this study are included in the article. Further inquiries can be directed to the corresponding authors.

## Abbreviations

The following abbreviations are used in this manuscript:

5-FU	5-Fluorouracil
ASHP	Association of Health-System Pharmacists
CSTD	Closed system transfer device
CV	Coefficient of variation
GC-MS	Gas chromatography coupled with mass spectrometry
HPLC-DAD	High-performance liquid chromatography coupled to a diode array detector
ICH	International Council for Harmonisation of Technical Requirements for Pharmaceuticals for Human Use
ISOPP	International Society of Oncology Pharmacy Practitioners
LOD	Limit of detection
LOQ	Limit of quantification
LC-MS/MS	Liquid chromatography coupled with tandem mass spectrometry
NIOSH	National Institute for Occupational Safety and Health
NCD	Noncommunicable diseases
RSD	Relative standard deviation
SmPC	Summary of product characteristics
UPLC-MS/MS	Ultra-performance liquid chromatography coupled with tandem mass spectrometry
